# Prevalence of 2009 Pandemic Influenza A (H1N1) Virus Antibodies, Tampa Bay Florida — November–December, 2009

**DOI:** 10.1371/journal.pone.0029301

**Published:** 2011-12-20

**Authors:** Chad M. Cox, Kate Goodin, Emily Fisher, Fatimah S. Dawood, Janet J. Hamilton, German F. Leparc, Monica Gray, Linda Nelson, Rebekah H. Borse, James A. Singleton, Carrie Reed, Amanda L. Balish, Jacqueline M. Katz, Richard S. Hopkins, Alicia M. Fry

**Affiliations:** 1 Epidemic Intelligence Service, Centers for Disease Control and Prevention (CDC), Atlanta, Georgia, United States of America; 2 National Center for Immunization and Respiratory Diseases (NCIRD), Centers for Disease Control and Prevention (CDC), Atlanta, Georgia, United States of America; 3 Bureau of Epidemiology, Florida Department of Health, Tallahassee, Florida, United States of America; 4 Epidemiology Elective Student, Centers for Disease Control and Prevention (CDC), Atlanta, Georgia, United States of America; 5 Florida Blood Services, St. Petersburg, Florida, United States of America; 6 All Children's Hospital, St. Petersburg, Florida, United States of America; 7 University of South Florida Pediatric Clinic, Tampa, Florida, United States of America; 8 National Center for Emerging and Zoonotic Infectious Diseases, Centers for Disease Control and Prevention (CDC), Atlanta, Georgia, United States of America; University of Hong Kong, Hong Kong

## Abstract

**Background:**

In 2009, a novel influenza virus (2009 pandemic influenza A (H1N1) virus (pH1N1)) caused significant disease in the United States. Most states, including Florida, experienced a large fall wave of disease from September through November, after which disease activity decreased substantially. We determined the prevalence of antibodies due to the pH1N1 virus in Florida after influenza activity had peaked and estimated the proportion of the population infected with pH1N1 virus during the pandemic.

**Methods:**

During November-December 2009, we collected leftover serum from a blood bank, a pediatric children's hospital and a pediatric outpatient clinic in Tampa Bay Florida. Serum was tested for pH1N1 virus antibodies using the hemagglutination-inhibition (HI) assay. HI titers ≥40 were considered seropositive. We adjusted seroprevalence results to account for previously established HI assay specificity and sensitivity and employed a simple statistical model to estimate the proportion of seropositivity due to pH1N1 virus infection and vaccination.

**Results:**

During the study time period, the overall seroprevalence in Tampa Bay, Florida was 25%, increasing to 30% after adjusting for HI assay sensitivity and specificity. We estimated that 5.9% of the population had vaccine-induced seropositivity while 25% had seropositivity secondary to pH1N1 virus infection. The highest cumulative incidence of pH1N1 virus infection was among children aged 5–17 years (53%) and young adults aged 18–24 years (47%), while adults aged ≥50 years had the lowest cumulative incidence (11–13%) of pH1N1 virus infection.

**Conclusions:**

After the peak of the fall wave of the pandemic, an estimated one quarter of the Tampa Bay population had been infected with the pH1N1 virus. Consistent with epidemiologic trends observed during the pandemic, the highest burdens of disease were among school-aged children and young adults.

## Introduction

The 2009 pandemic influenza A (H1N1) virus (pH1N1) was first identified in April 2009 and caused widespread illness in the United States and around the world [1]. The Centers for Disease Control and Prevention (CDC) estimated that during the pandemic, 14–29% of the US population had a clinical case of influenza [2]. However this estimate excluded subclinical cases which may have accounted for 24–36% of all infections [3], [4], [5], [6].

**Figure 1:**
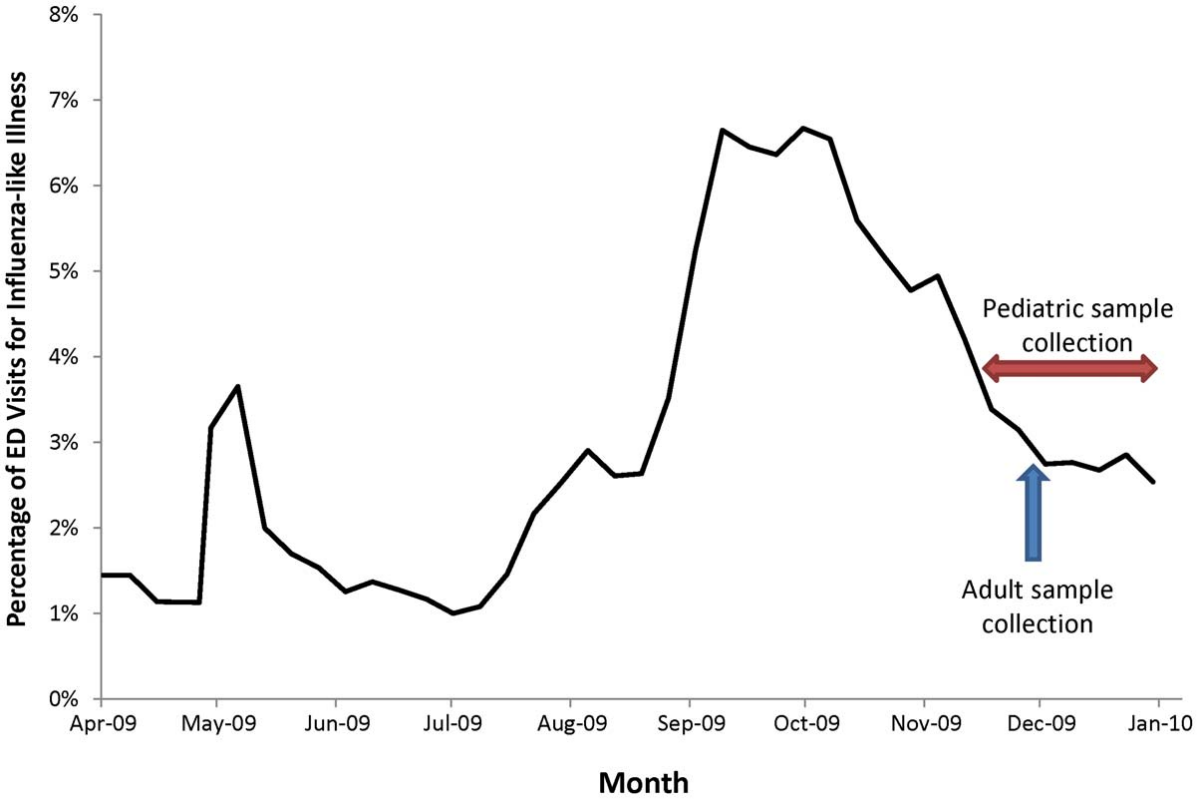
Percentage of Emergency Department (ED) visits for influenza-like illness (ILI)*, Florida Electronic Surveillance System for the Early Notification of Community-based Epidemic (ESSENCE), and time period of serum collection for seroprevalence survey —Tampa Bay Florida**— April 2009–January 2010. *Influenza-like illness (ILI) is defined as fever (≥100°F) accompanied by either cough or sore throat **Includes Hillsborough, Manatee, Pinellas and Pasco counties.

During the 2009 pandemic, Florida employed a surveillance system that tracked the percentage of Emergency Department (ED) visits for influenza-like illness (ILI) throughout the state. According to surveillance data, Tampa Bay experienced a gradual increase in influenza activity in the spring and summer of 2009, followed by a large fall wave of influenza activity that peaked in late October and decreased steadily thereafter (Figure 1). Estimating the total number of pH1N1 virus infections in Tampa Bay that were acquired during this time period presented several challenges. Existing disease surveillance likely provided an underestimate of the true proportion of individuals infected, due to its passive nature. In addition, patients with laboratory-confirmed infections represented only a fraction of the total burden, as not all infected persons sought medical care, were tested for influenza, or tested positive for influenza virus infection due to the timing or quality of the specimen collected.

Serosurveys, which estimate the prevalence of antibodies to a specific pathogen, can be a valuable tool in determining the proportion of the population infected with a novel virus. Unlike most influenza surveillance, which relies on presentation of clinical illness, serosurveys capture persons that experienced symptomatic or asymptomatic illness, and can provide information on total infections which may be underestimated with traditional surveillance methodologies. However, serosurveys are limited by the sensitivity and specificity of the assay employed to detect antibody titers [7] and by the presence of cross reactive antibodies from prior exposure to antigenically related viruses [8], [9]. Furthermore, assays to detect antibody against influenza viruses cannot distinguish between antibody elicited by virus infection versus vaccination.

To date, one published study has reported on the prevalence of pH1N1 antibodies among residents in one region of the United States [10]. Additional studies performed throughout the world have also been published, adding to the body of literature describing the disease burden of the pH1N1 pandemic [9], [11], [12], [13], [14], [15], [16], [17], [18], [19], [20], [21], [22], [23], [24]. The objective of our study was to determine the seroprevalence of pH1N1 antibodies among residents of Tampa Bay, Florida after the peak of the fall wave and prior to widespread vaccination.

## Methods

In November and December 2009, after pH1N1 virus activity in Tampa Bay had peaked (Figure 1), we collected a convenience sample of de-identified, leftover serum specimens (initially drawn for other laboratory testing) from residents of Pasco, Hillsborough, Manatee and Pinellas counties in Tampa Bay, Florida.

We sought to collect 160 specimens from each of six age groups: <5 years, 5–17 years, 18–24 years, 25–49 years, 50–64 years and ≥65 years. Infants less than 6 months were excluded due to the potential for maternal antibody transmission. The required sample size was calculated using relative standard error measurements. We estimated that the lowest seroprevalence for all groups would be among adults aged ≥65 years. At the time of the serosurvey, we estimated that 15% of this age group would be seropositive, requiring a sample size of approximately 160 to maintain a relative standard error less than 20%. We were able to collect at least 160 samples from all ages groups except for children aged <5 years for which we were only able to collect 60 samples. We estimated that the seroprevalence among this age group would be high (30%), and therefore despite the small sample size, would meet the relative standard error criteria of less than 20%.

This study was proposed to the Florida Department of Health Institutional Review Board (IRB) and Centers for Disease Control and Prevention (CDC) IRB who considered the investigation as public health response, and therefore not subject to IRB review and approval.

Leftover serum specimens for Tampa Bay residents aged ≥16 years were collected from a large blood bank testing facility during a 4-day period from November 30 -December 3, 2009 (Figure 1). For residents aged <16 years, leftover specimens were collected from a children's medical center and an outpatient pediatric clinic from November 14 to December 31, 2009. The majority of specimens collected from the children's medical center had been collected for allergy and immunology testing. Leftover specimens from the pediatric clinic had been originally collected for routine outpatient testing.

Antibodies against pH1N1 virus were detected by the hemagglutination-inhibition (HI) assay as previously described using A/California/07/2009 virus [25], [26]. All specimens were tested in triplicate. Specimens with a geometric mean HI titer ≥40 were considered to be seropositive. Total seroprevalence results were age-standardized based on Tampa Bay population estimates from the American Community Survey. Previous studies have shown that an HI titer ≥40 is associated with a ≥ 50% reduced risk of contracting seasonal influenza virus infection among susceptible persons [27], [28].

Using sera from patients with pH1N1 laboratory-confirmed infections and non-exposed United States residents, a previous study determined that a threshold HI titer ≥40 yielded a sensitivity of 75% and specificity of 97% in determining previous infection with the pH1N1 virus among persons <60 years of age [7]; specificity was shown to decrease to 94% among persons >60 years of age. We adjusted the overall seroprevalence results to account for both the sensitivity and specificity of the HI assay, terming the resultant estimate the assay-adjusted seroprevalence (Appendix S1).

Because serology cannot differentiate between antibodies produced by virus infection and response to vaccination, we developed a simple statistical model to estimate the proportion of seropositive results due to pH1N1 vaccination coverage (i.e., vaccine-induced seropositivity ) (Appendix S2). Monthly vaccination coverage estimates for the state of Florida during November and December 2009 were calculated based on combined Behavioral Risk Factors Surveillance System (BRFSS) and National 2009 H1N1 Flu Survey (NHFS) data [29], [30], [31], [32]. In the model we used vaccination coverage estimates, vaccine immunogenicity estimates from the literature [33], [34], [35], and an estimate of the proportion infected prior to vaccination to estimate the proportion with vaccine-induced seropositivity not infected prior to vaccination. To estimate the proportion of the population infected with the pH1N1 virus prior to the serosurvey, we subtracted this proportion from the seroprevalence estimate.

## Results

**Figure 2:**
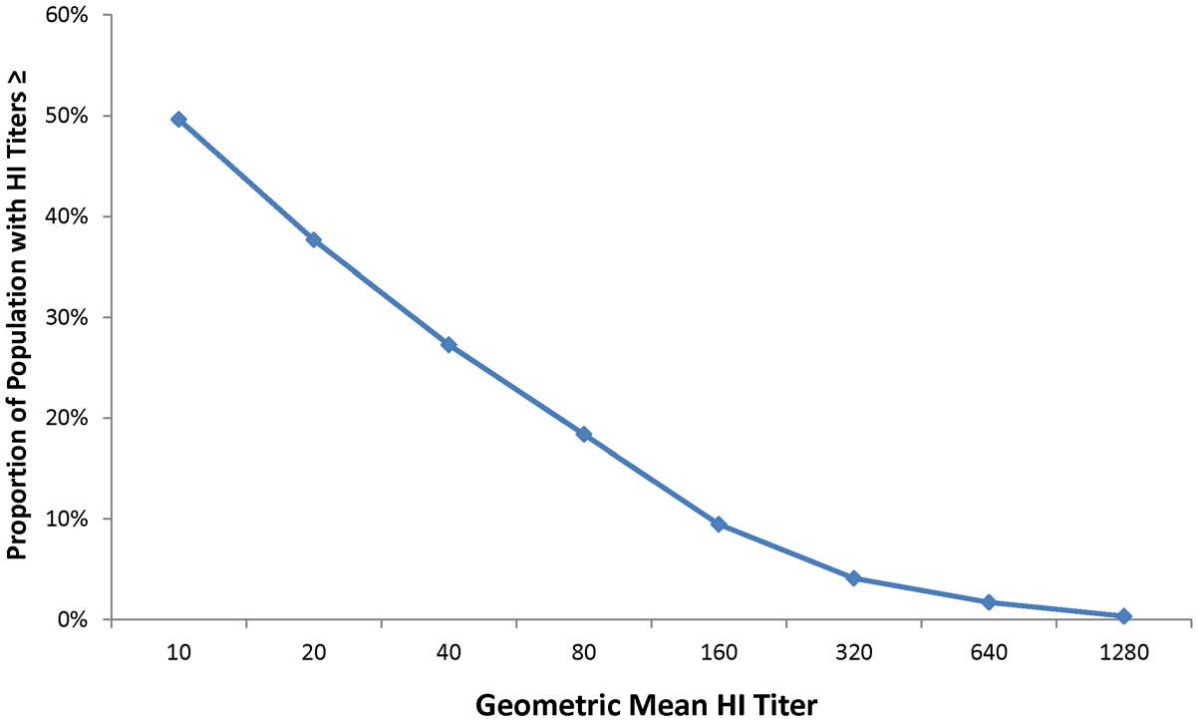
Reverse cumulative distribution curve of geometric mean HI titers for study samples, Tampa Bay, Florida – November-December 2009.

Overall, 27% of the study sample had pH1N1 antibody titers ≥40 with an age-standardized seroprevalence of 25% (Table 1). A cumulative reverse curve demonstrating the distribution of geometric mean titres (GMT) is shown in Figure 2. After adjusting for HI assay sensitivity and specificity, the overall assay-adjusted seroprevalence was 30%. The highest assay-adjusted seroprevalence was in children aged 5–17 years (60%) and young adults aged 18–24 years (50%). Older adults had the lowest assay-adjusted seroprevalance, ranging from 17–24%.

**Table 1:** Proportion of Tampa Bay population with elevated pH1N1 antibody titers and adjustment for hemagglutination inhibition (HI) assay sensitivity and specificity by age group — Tampa Bay Florida, November-December 2009.

Age Group	Number tested	HI titer ≥20 (n)	Prevalence of HI titer ≥20,% (95% CI)	HI titer ≥40 (n)	Prevalence of HI titer ≥40% (95% CI)	Assay-adjusted^1^ prevalence(HI titer ≥40)% (95% CI)
**<5 years**	60	20	33 (21–45)	17	28 (17–40)	35 (23–47)
**5–17 years**	159	78	49 (41–57)	73	46 (38–54)	60 (52–67)
**18–24 years**	150	74	49 (41–57)	59	39 (32–47)	50 (42–58)
**25–49 years**	169	56	33 (26–40)	34	20 (14–26)	24 (17–30)
**50–64 years**	173	46	27 (20–33)	27	16 (10–21)	18 (12–23)
**65+ years**	165	56	34 (27–41)	29	18 (12–23)	17 (11–22)
**Total**(Age-standardized )^2^	876	330	36 (33–39)	239	25 (22–28)	30 (27–34)

1 Seroprevalence adjusted for assay sensitivity and specificity. For children and adults aged <65 years, assay-adjusted seroprevalence was calculated using a sensitivity of 75% and a specificity of 97%. For adults aged ≥ 65 years, assay-adjusted seroprevalence was calculated using a sensitivity of 75% and a specificity of 94%.

2 Total seroprevalence results were age-standardized based on Tampa Bay population estimates from the American Community Survey (includes residents of Pasco, Hillsborough, Manatee and Pinellas counties).

The overall BRFSS/NHFS vaccination coverage estimate for Florida two weeks prior to the time of specimen collection was 9.0%, with the highest vaccination coverage among children aged <5 years (17%) and school-aged children aged 5–17 years (15%) (Table 2). Based on data from previously published studies, we assumed vaccine immunogenicity ranged from 60% in children aged <5 years to 95% in adults aged 18–64 years [33], [34], [35]. Using these figures, we estimated that 5.9% of the Tampa Bay population was seropositive due to vaccination, with the highest proportion among children <5 years (7.5%) and the lowest proportion among young adults aged 18–24 years (3.1%).

**Table 2:** Statistical model to estimate the proportion of Tampa Bay residents with vaccine-induced pH1N1 virus seropositivity in November- December 2009.

Age Group	Vaccine coverage estimate^1^	Vaccine immunogenicity estimate	Proportion with vaccine-induced seropositivity 2	Proportion with infection and vaccination^3^	Proportion with vaccine-induced seropositivity not infected prior to vaccination^4^
**<5 years**	17%	60%	10%	2.5%	7.5%
**5–17 years**	15%	80%	12%	5.8%	6.4%
**18–24 years**	6.0%	95%	5.7%	2.6%	3.1%
**25–49 years**	6.0%	95%	5.7%	1.0%	4.7%
**50–64 years**	7.4%	95%	7.0%	0.7%	6.3%
**≥65 years**	9.8%	85%	8.3%	0.7%	7.6%
**Total**	9.0%	85%	7.7%	1.7%	5.9%

1 Estimated from Behavioral Risk Factors Surveillance System (BRFSS) and National 2009 H1N1 Flu Survey (NHFS) for adult vaccination through mid-November and a weighted pediatric vaccination estimate for the two week period prior to specimen collection (November 1 – December 16, 2009).

2 Estimated proportion of population with vaccine-induced seropositivity (≥1∶40 GMT)  =  (vaccine coverage) x (proportion with ≥1∶40 seropositivity).

3 Estimated proportion with pH1N1 virus infection prior to vaccination  =  ([assay adjusted seroprevalence] minus [estimated proportion of population with vaccine-induced seropositivity]) x (estimated proportion of population with vaccine-induced seropositivity).

4 Proportion with vaccine-induced seropositivity not infected prior to vaccination  =  (estimated proportion of population with vaccine-induced seropositivity) minus (estimated proportion of population with pH1N1 virus infection prior to vaccination).

**Figure 3:**
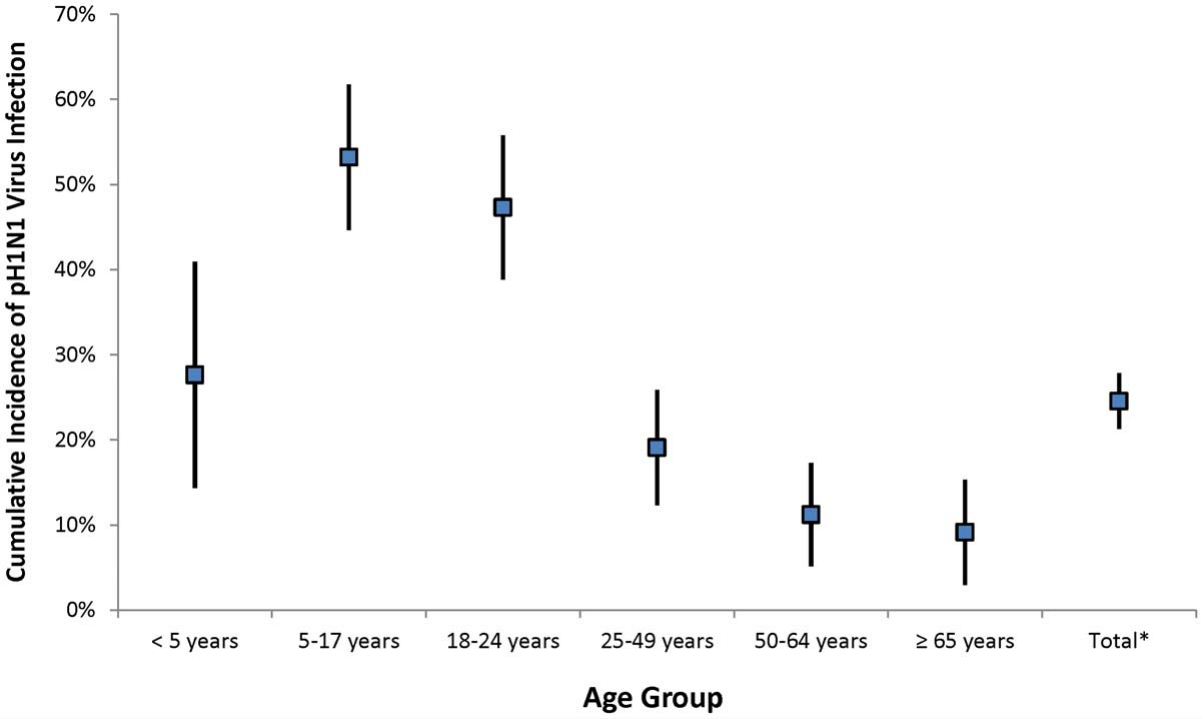
Estimated proportion of the population with pH1N1 virus infection — Tampa Bay, Florida – November-December 2009. *Estimate of total cumulative incidence is age-standardized.

The proportion of Tampa Bay residents that were estimated to be seropositive due to infection with pH1N1 virus after the peak of the fall wave was 25% (Figure 3). The highest cumulative incidence of infection with pH1N1 virus was among children aged 5–17 years and young adults aged 18–24 years (53% and 47%, respectively). Adults aged 50–64 years and ≥65 years had the lowest cumulative incidence of infection with the pH1N1 virus (11% and 9.2%, respectively). Applying these results to the Tampa Bay population, approximately 700,000 of Tampa Bay's 2.8 million residents were infected with the pH1N1 virus and 250,000 residents had received the pH1N1 vaccine by the end of the second wave of the pandemic.

## Discussion

By December 2009, an estimated 30% of Tampa Bay's population had elevated levels of antibodies against the pH1N1 virus (25% from infection and 5.9% from vaccination). Our estimates indicate that half of young adults aged 18–24 years and more than half of school-aged children had antibodies to the pH1N1 virus at titers of ≥40 at that time. Thus, the proportion of the Tampa Bay population among these age groups that remained susceptible to pH1N1 virus infection by December 2009 was markedly decreased.

Our seroprevalence estimates are similar to previously published studies from Pittsburgh, PA [10] and international studies that collected sera at comparable time periods [11], [13], [15], [16], [20], [24], [36]. Consistent with other studies, school-aged children were estimated to have had the highest seroprevalance of pH1N1 antibodies [10], [11], [12], [13], [15], [24]. This result coincides with the elevated clinical attack rates of pH1N1 illness observed among children during the pandemic and the focused vaccination campaigns for this age group [19], [37]. While pH1N1 vaccine supplies were limited, school-aged children were targeted for vaccination due to outbreaks of disease in settings such as schools [38], [39]. We found a similarly elevated seroprevalence among young adults aged 18–24 years, consistent with other studies which employed a comparable age distribution [9], [12], [13].

Though the availability of pH1N1 vaccine was still limited by the time of the serosurvey (52 million doses had been distributed in the United States by the end of November, enough to vaccinate 17% of the population), school-aged children comprised one of the target groups for initial vaccination campaigns, and thus were more likely to receive the vaccine earlier [39]. By two weeks prior to serum collection, an estimated 15% of school-aged children and 9% of the overall Florida population were vaccinated (Table S1). However, even after adjusting for vaccine-induced seropositivity, we found that a substantial proportion of school aged children (53%) and young adults (47%) had evidence of infection with pH1N1 virus. By May 2010, the proportion of the Florida population that had received the pH1N1 vaccine had increased to 22% [29]. Thus, it is likely that the proportion of the Tampa Bay population that was susceptible to the 2009 H1N1 virus at the beginning of the 2010–2011 influenza season was lower.

Our survey has several potential limitations. First, we did not have baseline serum specimens prior to the 2009 pandemic for comparison and therefore were not able to test for a four-fold rise in antibody titers or adjust for pre-existing, cross-reactive antibody titers. Previous studies have suggested that cross-reactive antibodies were most common among persons aged ≥60 years [9], [11]. Second, our serum specimens came from blood-bank and leftover laboratory testing; thus those sampled were not representative of the Tampa Bay population as a whole. Third, HI titers of ≥40 have been used as a threshold criteria for seropositivity by other studies and are correlated with immunity [27], [28]; however, lower antibody titers may occur in some people with evidence of polymerase chain reaction (PCR)-confirmed influenza infection [7]. While we did adjust for the sensitivity and specificity of the serologic assay, we still may have underreported the number of pH1N1 infections that occurred by December 2009 in Tampa Bay. Finally, because we used anonymous specimens for testing, we were not able to collect individual-level vaccination or symptom data. We sought to counter part of this limitation by using vaccination estimates specific to Florida. However, the vaccination data was not specific to the study area and was collected by surveys, and thus subject to non-response bias after weighting adjustments and recall error. Despite these limitations, our results were similar to surveys using other specimen sources [9], [11], [12], [13].

In summary, we performed a serosurvey in Tampa Bay to determine the prevalence of antibodies to the pH1N1 virus after the fall wave of the pandemic, and during the early phases of the pH1N1 vaccination campaign in Florida. We adjusted seroprevalence results to account for HI assay specificity and sensitivity and employed a simple statistical model to estimate the proportion of seropositivity due to pH1N1 virus infection and vaccination. Our results provide evidence for substantial immunity against the pH1N1 virus among the Tampa Bay population. Though disease activity decreased after December 2009, vaccination levels continued to increase in Florida; thus the proportion of the population with immunity to the pH1N1 virus by the end of the pandemic was probably higher than the estimates presented herein.

## Supporting Information

Appendix S1 **Method of seroprevalence adjustment to account for hemagglutination-inhibition (HI) assay sensitivity and specificity.**
(DOCX)

Appendix S2 **Simple statistical model used to estimate the proportion of seropositive results due to vaccination.**
(DOCX)

Table S1 **Statistical model to estimate the proportion of Tampa Bay residents with vaccine-induced pH1N1 virus seropositivity in November- December 2009, including all components and equations.**
1 Estimated from Behavioral Risk Factors Surveillance System (BRFSS) and National pH1N1 Flu Survey (NHFS)
2 Vaccine immunogenicity estimates based on published immunogenicity studies [27], [28], [29]
3 Estimated proportion of population with vaccine-induced seropositivity (≥1∶40 GMT)  =  (vaccine coverage) x (proportion with ≥1∶40 antibody response)
4 Seroprevalence adjusted for assay sensitivity and specificity. For children and adults aged <65 years, assay-adjusted seroprevalence was calculated using a sensitivity of 75% and a specificity of 97%. For adults aged ≥ 65 years, assay-adjusted seroprevalence was calculated using a sensitivity of 75% and a specificity of 94% [7]
5 Estimated proportion with pH1N1 virus infection prior to vaccination  =  ([assay adjusted seroprevalence] minus [estimated proportion of population with vaccine-induced seropositivity]) x (estimated proportion of population with vaccine-induced seropositivity)
6 Proportion with vaccine-induced seropositivity not infected prior to vaccination  =  (estimated proportion of population with vaccine-induced seropositivity) minus (estimated proportion of population with pH1N1 virus infection prior to vaccination)
7 Proportion infected with pH1N1 virus  =  (assay-adjusted seroprevalence) minus (proportion with vaccine-induced seropositivity not infected prior to vaccination).
(DOCX)
