# Supplementary material for: Prevalence of 2009 Pandemic Influenza A (H1N1) Virus Antibodies, Tampa Bay Florida — November–December, 2009
Source: PLoS One. 2011 Dec 20;6(12):e29301. doi: 10.1371/journal.pone.0029301 (PMC3243696; doi:10.1371/journal.pone.0029301)
Supplement: Appendix S1 — Method of seroprevalence adjustment to account for hemagglutination-inhibition (HI) assay sensitivity and specificity. (DOCX) [file pone.0029301.s001.docx]

**Appendix S1**

**Method of seroprevalence adjustment to account for hemagglutination-inhibition (HI) assay sensitivity and specificity**

To adjust seroprevalence results to account for both the sensitivity and specificity of the HI assay, we applied the known and unknown variables to a 2 x 2 matrix (below) where x= total number of true 2009 pandemic influenza A H1N1 (pH1N1) positives, and N= total number tested.

|  |  | **pH1N1 Virus Infection** | |  |
| --- | --- | --- | --- | --- |
|  |  | **+** | **—** |  |
| **HI Testing** | **+** | 0.75x | 0.03(N-x) | Total with positive HI Test |
|  | **—** | 0.25x | 0.97(N-x) | Total with negative HI Test |
|  |  | x | N-x | N |

We used an algebraic expression to describe the relationship among those with positive HI testing:

0.75x + 0.03(N-x) = Total with positive HI test.

Reducing the equation to solve for x yields the expression below.

x = (Total with positive HI test – 0.03N)/0.72

The adjusted seroprevalence was calculated by dividing the total number of true positives (x) by the total number tested (N). For children and adults aged <65 years, assay-adjusted seroprevalence was calculated using an assay sensitivity of 75% and a specificity of 97% [[7](file:///\\cdc.gov\private\M130\cyv5\Influenza\Domestic\Florida\Manuscript\PLOS%20Submission\Revision%20Submission%2011-18-11\Florida%20Seroprevalence%20Study_clean.docx#_ENREF_7)]. For adults aged ≥ 65 years, assay-adjusted seroprevalence was calculated using an assay sensitivity of 75% and a specificity of 94%. For our estimations, we used assay sensitivities reported for adults > 60 years of age for adults ≥ 65 years of age and assumed all adults aged 50-64 years had assay sensitivities similar to those < 60 years of age [[7](file:///\\cdc.gov\private\M130\cyv5\Influenza\Domestic\Florida\Manuscript\PLOS%20Submission\Revision%20Submission%2011-18-11\Florida%20Seroprevalence%20Study_clean.docx#_ENREF_7)].
