# Supplementary material for: Prevalence of 2009 Pandemic Influenza A (H1N1) Virus Antibodies, Tampa Bay Florida — November–December, 2009
Source: PLoS One. 2011 Dec 20;6(12):e29301. doi: 10.1371/journal.pone.0029301 (PMC3243696; doi:10.1371/journal.pone.0029301)
Supplement: Appendix S2 — Simple statistical model used to estimate the proportion of seropositive results due to vaccination. (DOCX) [file pone.0029301.s002.docx]

**Appendix S2**

**Simple statistical model used to estimate the proportion of seropositive results due to vaccination**

We created a simple statistical model to estimate the proportion of seropositive results due to vaccination (i.e., vaccine-induced seropositivity). Inputs to the model included vaccination coverage estimates, vaccine immunogenicity estimates from the literature, and an estimate of the number of persons vaccinated who had pH1N1 virus infection prior to vaccination.

Using the weekly rate of increase in vaccination coverage from national NHFS estimates, we interpolated weekly Florida vaccination coverage estimates from the monthly Florida NHFS/BRFSS estimates. We used vaccination estimates for two weeks prior to the date of sample collection to account for the delay in immune response to vaccination [[40](file:///\\cdc.gov\private\M130\cyv5\Influenza\Domestic\Florida\Manuscript\PLOS%20Submission\Revision%20Submission%2011-18-11\Florida%20Seroprevalence%20Study_clean.docx#_ENREF_40)]. Vaccination estimates for adults correspond to the week of November 16-22, 2009, two weeks prior to the sample collection period of November 30 through December 3, 2009 (Table S1).

Pediatric samples were collected over 7 weeks (November 14 through December 31, 2009) with a greater proportion of samples collected earlier in that time period. We grouped pediatric samples by week of sample collection. Vaccination estimates two weeks prior to sample collection were then applied to each group. We then calculated an overall weighted vaccination estimate based on the weekly vaccination estimates and the number of children sampled each week (**column A**).

We assumed that not all vaccinated individuals developed an HI antibody titer of ≥40 after vaccination. Our model included an estimate of vaccine immunogenicity by age group (**column B**) based on pH1N1 vaccine trials among various age groups [[33](file:///\\cdc.gov\private\M130\cyv5\Influenza\Domestic\Florida\Manuscript\PLOS%20Submission\Revision%20Submission%2011-18-11\Florida%20Seroprevalence%20Study_clean.docx#_ENREF_33),[34](file:///\\cdc.gov\private\M130\cyv5\Influenza\Domestic\Florida\Manuscript\PLOS%20Submission\Revision%20Submission%2011-18-11\Florida%20Seroprevalence%20Study_clean.docx#_ENREF_34),[35](file:///\\cdc.gov\private\M130\cyv5\Influenza\Domestic\Florida\Manuscript\PLOS%20Submission\Revision%20Submission%2011-18-11\Florida%20Seroprevalence%20Study_clean.docx#_ENREF_35)]. We multiplied the “vaccine coverage estimate” (**column A**) by the “vaccine immunogenicity estimate” (**column B**) to arrive at a product termed “proportion with vaccine-induced seropositivity” (**column C**).

In order to account for individuals who may have been infected with pH1N1 virus prior to vaccination, we first subtracted the “proportion with vaccine-induced seropositivity” (**column C**) from the assay-adjusted seroprevalence estimate (**column D**) to arrive at an adjusted seroprevalence estimate that excludes vaccine-induced seropositivity. We assumed that this proportion of people may have had pH1N1 virus infection prior to vaccination, thus we multiplied this number by the “proportion with vaccine-induced seropositivity” (**column C**) to calculate the resulting “proportion with infection prior to vaccination” (**column E**). The same calculation was applied based on the lower and upper bounds of the confidence intervals from the assay-adjusted seroprevalance to arrive at minimum and maximum estimates of the “proportion with infection prior to vaccination”.

The “proportion with vaccine-induced seropositivity not infected prior to vaccination” (**column F**) was calculated by subtracting the “proportion with infection prior to vaccination” (**column E**) from the “proportion with vaccine-induced seropositivity” (**column C**). We calculated a maximum around this estimate by subtracting the minimum value of the “proportion with infection prior to vaccination” from the “proportion with vaccine-induced seropositivity ” (**column C**). Likewise we calculated a minimum around the estimate by subtracting the maximum value of the “proportion with infection prior to vaccination” from the “proportion with vaccine-induced seropositivity” (**column C**).

We calculated the “proportion infected with pH1N1 virus” (**column G**) by subtracting the “proportion with vaccine-induced seropositivity not infected prior to vaccination” (**column F**) from the overall assay-adjusted seroprevalence (**column D**). We calculated a maximum around this estimate by subtracting the minimum value of the “proportion with vaccine-induced seropositivity not infected prior to vaccination” from the upper confidence interval of the assay-adjusted seroprevalence. A minimum was calculated by subtracting the maximum value of the “proportion with vaccine-induced seropositivity not infected prior to vaccination” from the lower confidence interval of the assay-adjusted seroprevalence.
