# Supplementary material for: Prevalence of 2009 Pandemic Influenza A (H1N1) Virus Antibodies, Tampa Bay Florida — November–December, 2009
Source: PLoS One. 2011 Dec 20;6(12):e29301. doi: 10.1371/journal.pone.0029301 (PMC3243696; doi:10.1371/journal.pone.0029301)
Supplement: Table S1 — Statistical model to estimate the proportion of Tampa Bay residents with vaccine-induced pH1N1 virus seropositivity in November- December 2009, including all components and equations. 1 Estimated from Behavioral Risk Factors Surveillance System (BRFSS) and National pH1N1 Flu Survey (NHFS) 2 Vaccine immunogenicity estimates based on published immunogenicity studies [27], [28], [29] 3 Estimated proportion of population with vaccine-induced seropositivity (≥1∶40 GMT) = (vaccine coverage) x (proportion with ≥1∶40 antibody response) 4 Seroprevalence adjusted for assay sensitivity and specificity. For children and adults aged <65 years, assay-adjusted seroprevalence was calculated using a sensitivity of 75% and a specificity of 97%. For adults aged ≥ 65 years, assay-adjusted seroprevalence was calculated using a sensitivity of 75% and a specificity of 94% [7] 5 Estimated proportion with pH1N1 virus infection prior to vaccination = ([assay adjusted seroprevalence] minus [estimated proportion of population with vaccine-induced seropositivity]) x (estimated proportion of population with vaccine-induced seropositivity) 6 Proportion with vaccine-induced seropositivity not infected prior to vaccination = (estimated proportion of population with vaccine-induced seropositivity) minus (estimated proportion of population with pH1N1 virus infection prior to vaccination) 7 Proportion infected with pH1N1 virus = (assay-adjusted seroprevalence) minus (proportion with vaccine-induced seropositivity not infected prior to vaccination). (DOCX) [file pone.0029301.s003.docx]

**Table S1.** Statistical model to estimate the proportion of Tampa Bay residents with vaccine-induced pH1N1 virus seropositivity in November- December 2009, including all components and equations

|  | **A** | **B** | **C**  **A*B** | **D** | **E**  **C*(D-C)** | **F**  **(C-E)** | **G**  **(D-F)** |
| --- | --- | --- | --- | --- | --- | --- | --- |
| **Age Group** | Vaccine coverage estimate^1^ (%) | Vaccine immunogenicity estimate^2^ (%) | Proportion with vaccine-induced seropositivity^3^ (%) | Assay-Adjusted Seroprevalence^4^  (%) | Proportion with infection and vaccination^5^ (%) | Proportion with vaccine-induced seropositivity not infected prior to vaccination^6^ (%) | Proportion infected with pH1N1 virus^7^,  % (Min-Max) |
| **< 5 years** | 17% | 60% | 10% | 35 (23-47) | 2.5% | 7.5% | 28 (14-41) |
| **5-17 years** | 15% | 80% | 12% | 60 (52-67) | 5.8% | 6.4% | 53 (45-62) |
| **18-24 years** | 6.0% | 95% | 5.7% | 50 (42-58) | 2.6% | 3.1% | 47 (39-56) |
| **25-49 years** | 6.0% | 95% | 5.7% | 24 (17-30) | 1.0% | 4.7% | 19 (12-26) |
| **50-64 years** | 7.4% | 95% | 7.0% | 18 (12-23) | 0.7% | 6.3% | 11 (5-17) |
| **≥ 65 years** | 9.8% | 85% | 8.3% | 17 (11-22) | 0.7% | 7.6% | 9.2 (3.0-15) |
| **Total** | 9.0% | 85% | 7.7% | 30 (27-34) | 1.7% | 5.9% | 25 (21-28) |
